# Pooling and Comparing Noise Annoyance Scores and “High Annoyance” (HA) Responses on the 5-Point and 11-Point Scales: Principles and Practical Advice

**DOI:** 10.3390/ijerph18147339

**Published:** 2021-07-09

**Authors:** Mark Brink, Lise Giorgis-Allemand, Dirk Schreckenberg, Anne-Sophie Evrard

**Affiliations:** 1 Federal Office for the Environment, CH-3003 Bern, Switzerland; 2 UMRESTTE UMR_T9405, IFSTTAR, Univ. Gustave Eiffel, Univ. Lyon, F-69675 Lyon, France; lise.giorgis-allemand@univ-eiffel.fr (L.G.-A.); anne-sophie.evrard@univ-eiffel.fr (A.-S.E.); 3 ZEUS GmbH, D-58095 Hagen, Germany; schreckenberg@zeusgmbh.de

**Keywords:** transportation noise, highly annoyed, exposure-response relationship, pooled analysis, conversion rules

## Abstract

The use of different noise annoyance scales across studies and socio-acoustic surveys, in particular the popular 5-point verbal and 11-point numerical scales, has made the evaluation, comparison, and pooling of noise annoyance responses among studies a taxing issue. This is particularly the case when “high annoyance” (HA) responses need to be compared and when the original studies used different scales; thus, there are different so-called cutoff points that define the part of the scale that indicates the HA status. This paper provides practical guidance on pooling and comparing the respective annoyance data in both the linear and logistic regression context in a statistically adequate manner. It caters to researchers who want to carry out pooled analyses on annoyance data that have been collected on different scales or need to compare exposure–HA relationships between the 5-point and 11-point scales. The necessary simulation of a cutoff point non-native to an original scale can be achieved with a random assignment approach, which is exemplified in the paper using original response data from a range of recent noise annoyance surveys. A code example in the R language is provided for easy implementation of the pertinent procedures with one’s own survey data. Lastly, the not insignificant limitations of combining and/or comparing responses from different noise annoyance scales are discussed.

## 1. Introduction

Noise annoyance can be defined as a multifaceted cognitive, affective, and behavioral response to noise [1]. As such, annoyance can be observed in social surveys as a retrospective judgment. It is typically measured by self-assessment with standardized questionnaire items. Several types of psychometric scales to measure the intensity of annoyance have emerged so far, some more, some less popular.

In noise annoyance surveys, two scales have become increasingly popular: the (so-called) 5-point verbal and the 11-point numerical annoyance scales that were recommended by the International Commission on Biological Effects of Noise (ICBEN) in 2001 [2]. The 5-point verbal ICBEN scale uses the scale points “not at all,” “slightly,” “moderately,” “very,” and “extremely,” marking clear semantic distinctions and roughly equidistant [2] from each other. The 11-point numerical scale ranges from the scale point value 0 (labeled “not at all”) to 10 (labeled “extremely”). The ISO has adopted large parts of the ICBEN’s original recommendation and recently published a revision of their standard on the assessment of noise annoyance using the very same scales [3]. To understand the popularity of these scales, Table 1 shows how often one or the other or both scales have been used in the 57 surveys included in the evidence review paper on noise annoyance, carried out by Guski et al. [1] for the recent WHO Environmental Noise Guidelines [4].

In addition to the question stem and scale characteristics themselves, detailed in [2,3], a de facto standard has emerged as to which degree of intensity of annoyance should be considered “high annoyance” or “highly annoyed” (HA), respectively, as indicated by choice of an alternative answer on these scales. Concerning the 5-point verbal scale, ICBEN’s recommendation is to use the upper two categories (the verbal marks “very” and “extremely”) as indicators of “high annoyance.” This corresponds to a cutoff point at 60% of the total scale length. No recommendation is provided for the 11-point scale, however, according to common practice, the upper three points on the numerical scale (8, 9, 10) are regarded as indicating “high annoyance” in the respondent. In this case, the cutoff point lies at 72.73% (cf. [5]). There are other scales (and cutoff points) in use as well, however, these are less common (see Table 1).

The widespread use of different scales and different cutoff points has made the pooling and comparison of noise annoyance responses a problematic issue. At the same time, there is a clear demand for up-to-date generalized exposure–effect relationships, as has been demonstrated, for example, in the scope of recent WHO work on noise effects [4].

The aim of the present paper is, therefore, to provide researchers guidance as regards the handling of different annoyance scales when aggregating annoyance response data from several studies for the purpose of comparisons or pooled analyses. In the first part of this paper, the scope lies on the conversion of original scale points into converted values on a unified 0–100 scale, suitable for linear regression analysis or in a purely descriptive context. In the second statistically and computationally more complex part, it will be demonstrated how to pool and compare logistic exposure-response relationships for %HA in which the original surveys used different scales and, hence, different cutoff points for the definition of HA.

## 2. Linear Regression Context: Conversion of Equidistant Verbal and Numerical Annoyance Scales to a Common Scale

To carry out absolute numerical comparisons between response values from different scales or to pool response values obtained from different scales, the original responses need to be converted and aligned to a common unified scale. Miedema and Vos [6] proposed such a common scale to run from 0 to 100. The choice of the values 0 and 100 for the lower and upper limits of this common scale is arbitrary but does not affect the converted values except for a scaling factor. However, the appropriate “rule” to convert any verbal or numeric original scale value to a value between 0 and 100 is not inherently obvious, even if the original scale can be regarded as an interval scale, i.e., having equally spaced scale points.

In the following, we will concentrate on the conversion of the 5-point and 11-point scales, whereby the below exercise applies in the same way to all types of scales. A numerical conversion to scale values between 0 and 100 assumes the original scales to be equidistant interval scales and that the first and last scale point on the 5-point or similar scale (“not at all” and “extremely”) and the scale points “0” and “10” on the 11-point scale represent the poles of the same annoyance intensity continuum that ranges between minimal (i.e., inexistent) and maximum (i.e., extreme) annoyance. Furthermore, we assume that the verbal scale point labels on the 5-point verbal ICBEN scale can be treated as representing equidistant intervals of annoyance intensity and henceforth can be projected without information loss on an interval scale with the values 0 = “not at all,” 1 = “slightly,” 2 = “moderately,” 3 = “very,” and 4 = “extremely,” We are aware that some issues surrounding the choice/assignment of a numerical value for verbal scale point labels on the 5-point verbal scale have not been fully resolved, but we will not discuss the issue any further; for a more thorough disquisition, see [2]. For the 11-point numerical scale from 0 to 10, equidistance and interval scale properties can be taken for granted.

One problem when converting discrete scale point values to values on the 0–100 scale is that it is basically unknown whether respondents interpret the point labels (figures or text) on the original scale as a descriptor of a discrete point or as a midpoint, or lower or upper boundary of a category that occupies an equal amount of the scale’s total length. Thus, under the above assumptions, four conversion types seem to be feasible: (a) the upscaling conversion in which each scale point value is multiplied with a constant, and the (b) lower bound, (c) midpoint, and (d) upper bound (of a response category) conversions. Table 2 shows the corresponding values for 4-, 5-, 6-, 7-, and 11-point scales. For reasons of comparability, the assigned “numeric value” in Table 2 always starts with the value 0 at the lowest scale point. The value 0 has a conceptually unambiguous meaning and clearly expresses that one is not annoyed at all.

**Figure 1:**
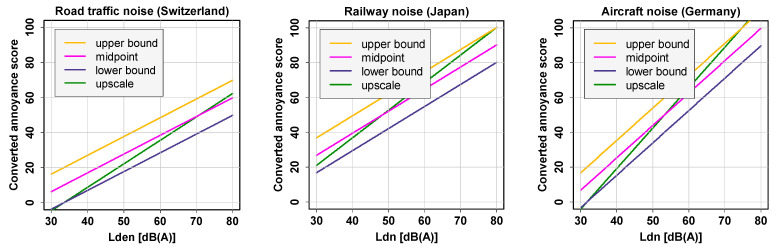
Examples of linear regression lines resulting from modeling with four different scale conversions of the 5-point scale, as listed in Table 2: **Left,** road traffic noise in Switzerland (whole country) [7]; **center,** railway noise in Japan (Sapporo) [8]; **right,** aircraft noise in Germany (Frankfurt) [9].

One now may ask which of the above-listed conversions to use in a specific case. Figure 1 can help to reach a decision, as it shows the exposure–annoyance relationships for the four different conversions plotted in the same graph. Each of the three graphs shows data from a different study and different noise source (road traffic, [7]; railway, [8]; aircraft, [9]) in which the converted annoyance score on the 5-point verbal scale was regressed on the noise metrics Lden (Day–evening–night level, with 5 and 10 dB penalties) or Ldn (Day–night level, with 10 dB penalty), respectively.

Clearly and of course expectedly, the lower bound, upper bound, and midpoint conversions just shift the linear regression lines along the y-axis, while the upscaling conversion (green regression lines) produces steeper slopes. This is due to the fact that this is the only conversion that uses the full 0–100 range to reflect ratings on the original scale, which also implies that it is the only conversion that keeps the slope that is observed with the original data. For the 5-point scale, multiplying its numeric value (0, 1, 2, 3, or 4) by 25 also allows upscaling to values expressible as integers while not only preserving equidistance between scale points but also preserving the zero (0) anchor point. However, the upscale conversion may overestimate the annoyance intensity slightly at the highest point (verbal mark “extremely”), as probably not all “extremely” annoyed persons would put themselves at exactly 100 on an underlying 0–100 intensity scale (cf. [10]). Similarly, the upscaling may lead to a slight underestimation of the annoyance at the lower end of the scale. Despite these weaknesses, we advocate to basically use the upscale conversion for linear regression or for descriptive purposes due to the advantages discussed above.

## 3. Logistic Regression Context: Simulating an Exposure–Response Relationship for the Percentage “Highly Annoyed” (HA) according to a Specified Cutoff Point

Noise abatement policies, e.g., the setting of exposure limits, rely to a large degree on exposure-response relationships that express the percentage highly annoyed (%HA) as a function of exposure. The focus on *highly* annoyed persons has many practical advantages compared to using annoyance score values, as was convincingly discussed by Schultz in [5] decades ago. However, when pooling or comparing original data that involve dichotomized categories of HA responses (0 or 1) as the response variable but which are based on different scales and cutoff points, it is not immediately obvious how to treat the responses in a statistically acceptable manner. Instead of just linearly converting scale values from one into another, such as what was described in the previous section, a slightly more complex approach is necessary. It basically involves three steps:

First, a common cutoff point, i.e. a percentage of the scale, needs to be defined based on which responses on the two (different) scales should be compared. The cutoff point defines which (upper) part of the annoyance scale reflects a HA response. A common cutoff to determine %HA is needed in those cases in which (1) one study or one given exposure–HA relationship needs to be compared to another that has a different HA cutoff, or (2) in pooled analyses of original survey response data, in which researchers want to combine the original responses from different studies that involve different annoyance scales to derive a common exposure–HA relationship.

Second, if the common cutoff point falls within a response category, the fraction of respondents below (F_below_) or above (F_above_) that common cutoff point within the respective category needs to be determined.

Third, based on an assignment of the binary response 1 (HA = 1) to a randomly chosen fraction of respondents above F_above_ and assignment of the response 0 (HA = 0) to the fraction below this value, a logistic exposure–HA relationship can be established that accounts for the common cutoff point. The necessary steps to do so are detailed in the next section.

### 3.1. Choice of a Common Cutoff Point and Determination of the Fractions of Responses above That Cutoff Point

According to the proposition of Schultz, respondents who choose scale points encompassing the upper 27–29% of a (numerical) annoyance scale, i.e., with a cutoff of about 72% of the scale, should be regarded as “highly annoyed” (HA). This cutoff more or less corresponds to choosing one of the two uppermost points on a 7-point scale or one of the three uppermost points on an 11-point scale. While the 72% cutoff point has been retained by Miedema and Oudshoorn [11] in their meta-analysis, the ICBEN recommended defining HA as those respondents who choose the upper two scale points “very” and “extremely” on a 5-point verbal scale, i.e., the upper 40% of the total scale length, which gives a cutoff point at 60%.

**Figure 2:**
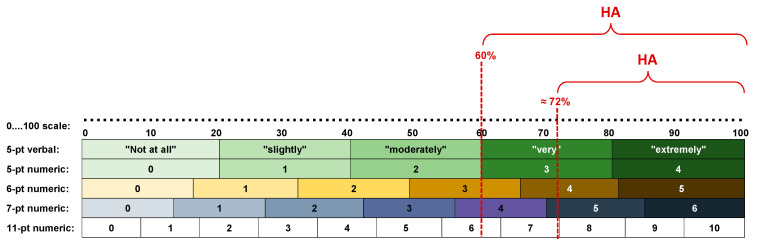
Commonly used noise annoyance scales and location of the most commonly used cutoff points 60% and ≈72% (red dashed lines) that are used to define a “highly annoyed” (HA) response.

Figure 2 reveals that the cutoff points 60% and 72% often fall *within* a response category (with the exception of the 60% cutoff point to determine HA on the 5-point verbal ICBEN scale).

A cutoff point on an annoyance scale above which respondents are considered HA is basically an arbitrary setting. The exact cutoff point x for a given number of scale points indicating “high annoyance” and a given total number of scale points of a scale can be calculated as

(1) $$x=1-\frac{\mathrm{Number}\;\mathrm{of}\;\mathrm{upper}\;\mathrm{scale}\;\mathrm{points}\;\mathrm{reflecting}\;“\mathrm{high}\;\mathrm{annoyance}”}{\mathrm{Total}\;\mathrm{number}\;\mathrm{of}\;\mathrm{points}\;\mathrm{on}\;\mathrm{the}\;\mathrm{scale}}$$

Many authors of more recent annoyance surveys want to compare their annoyance curves (based on whatever original scale) with the older so-called “EU/Miedema” curves [11] upon which the EU’s noise abatement policy was based for many years. These curves are based on a cutoff point of 72%. For the 5-point verbal scale with the scale points 3 (“very”) and 4 (“extremely”) (the ICBEN recommendation), the corresponding cutoff point is 60%. To compare the latter to the former, e.g., when only the 5-point scale has been used in a survey, the response data must be “tweaked” in a way to mimic a 72% cutoff point. Currently, with the widespread acceptance of the ICBEN recommendation [2], most responses from the 11- and 5-point scales need to be pooled or compared to each other. This means that the relevant cutoff point is, in fact, not 72% but 72.73%, i.e., 1−(3/11), according to Equation (1). For the remainder of this paper, we will work with this example/figure.

If the cutoff point lies *within* the category chosen by the respondent, it is not known whether this is a response below or above the cutoff point. Assuming that the distribution of the annoyance intensity within a category is uniform, one can calculate the theoretically expected fraction of responses in that category to be below (F_below_) or above (F_above_) the cutoff point by using Equation (2).

(2) $$F_{\mathrm{below}}=1-\frac{U-x}{U-L};\;F_{\mathrm{above}}=\frac{U-x}{U-L}$$

where:

F_below_, F_above,_ fraction of responses above or below the cutoff point;

x, cutoff point;

L, lower bound of category;

U, upper bound of category.

**Figure 3:**
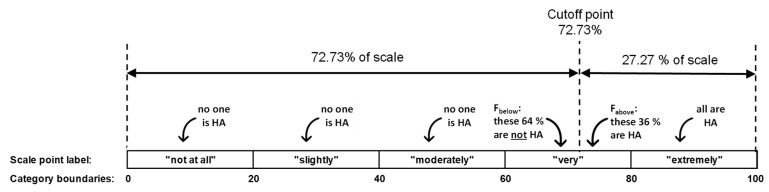
Cutoff point of 72.73% (which corresponds to the three uppermost scale points 8, 9, 10 on the 11-point scale) on the 5-point scale and assigned percentage of HA within the five categories “not at all,” “slightly,” “moderately,” “very,” “extremely.”

As an example, for the 5-point scale, given L = 60%, U = 80%, and x = 72.73%, F_below_ would be 0.64 and F_above_ = 0.36. While Figure 3 below gives an illustration of the pertinent fractions in this example, the respective values for other scale/cutoff point combinations are listed in Table 3.

It is important to note that F_above_ in Table 3 can also and directly be used as the value for a “weighted” HA response given the respective cutoff point. If, e.g., a respondent in a survey marked the answer “very” on the 5-point scale, instead of “extremely,” this response would only count as HA = 0.36 (for a cutoff point of 72.73%) or HA = 0.4 (for a cutoff point of 72%), respectively, instead of HA = 1. Such weighted responses can be used for merely descriptive analyses and frequency tables, such as counting the number of HA per exposure category etc., but not as a statistical weight for weighted logistic regression analysis, which seems to be a frequent misconception.

**Figure 4:**
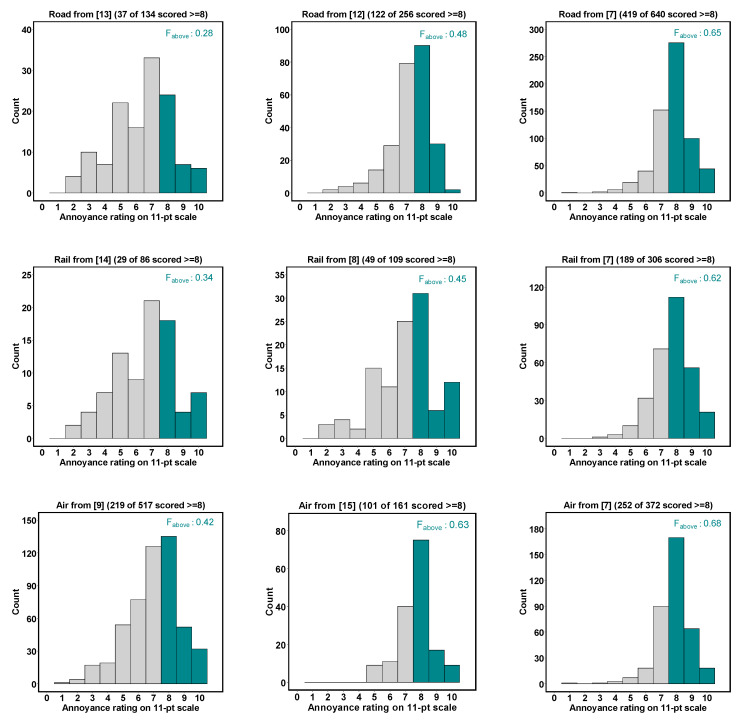
Distribution of responses on the 11-point scale of respondents that chose “very” (numerical value 3) on the 5-point scale in studies on road traffic, railway, and aircraft noise, with the fraction above the cutoff point of 72.73% in dark green. Data sources are from the following studies: [7,8,9,12,13,14,15].

The theoretical approach discussed above resides on the idea that, on average, *exactly* 36% of respondents that score “very” on the 5-point scale would choose a value equal to or greater than 8 on the 11-point scale (and 64% of them a value below 8). However, the empirical value may deviate from 36% for yet unknown reasons. To arrive at an empirically more solid value for F_above_, the basic question to ask is, “How high is the fraction of ‘very’ (on the 5-point scale) annoyed respondents that score 8, 9, or 10 on the 11-point scale?” To shed some light on this, Figure 4 shows the frequency distribution of the answers on the 11-point scale for those respondents that chose “very” on the 5-point scale in a collection of independent surveys for which we obtained the response data from *both* the 5-point and 11-point scales. In each histogram in Figure 4, the fraction of respondents above the cutoff point of 72.73% is colored in dark green.

From Figure 4, one can learn that in the majority of the studies at hand, F_above_ is, in fact, larger than 0.36. In the above (not necessarily representative) sample of studies, the average F_above_ is about 0.5. Indeed, a robust estimate of F_above_ is crucial for simulating a cutoff point of 72.73% with responses on the 5-point scale, as will be discussed in the next section.

### 3.2. Determination of the Exposure–Response Relationship for %HA for an Arbitrary Cutoff Point

In the following, we consider the case in which one has response data on the 5-point ICBEN scale but not on the 11-point scale and wants to simulate a cutoff point of 72.73%. This is to produce an exposure-response model (or curve) that is “compatible” with the 72.73% cutoff point on the 11-point scale (and, hence, ~compatible with the so-called “EU curve” [16]) or, in other words, can “mimic” the non-native to the scale cutoff point of 72.73%. This represents the most frequent problem to solve in our view, as the 11-point scale is used about twice as often as the 5-point scale (cf. Table 1). This case also accounts for the most often-adopted cutoff value of 72.73% (corresponding to 73% or 72% if rounded up or down).

As logistic regression allows only for 1 or 0 as the response value, it is not immediately obvious how to handle responses whose corresponding category (here, the “very” category) encloses the cutoff point (cf. Figure 3). This means that some of the respondents that chose “very” on the 5-point scale are below, while some are above the 72.73% cutoff point. Among the ones choosing 3 = “very,” the fraction expressed in the figure F_above_ can be considered HA (HA = 1) (cf. Equation (1)). Consequently, the fraction expressed in the figure F_below_ is not considered HA (HA = 0).

The imminent question now is how to estimate an exposure-response relationship for the probability to be HA, for a cutoff point of 72.73%, with response data on the 5-point scale (which does not have a “scale-inherent” cutoff point at 72.73%). To do this, we propose a simulation approach to estimate the desired exposure-response relationship from many subsamples. The key element here is the assignment of the HA = 1 status to a randomly sampled fraction (namely F_above_) of respondents that scored “very” on the 5-point scale. The procedure can be implemented in the following steps:

1. From the data table containing exposure and response data from the 5-point scale, create a subtable with only those respondents that have values 0, 1, 2, or 4 on the 5-point verbal scale. Assign the binary value HA = 0 to the responses 0, 1, 2, and the value HA = 1 to response 4.
2. Create a second subtable containing only the cases with value 3 (“very”) on the 5-point scale.
3. Randomly sample a fraction of F_above_ cases in that second subtable and assign these cases the binary value HA = 1, and the remaining cases a value of HA = 0.
4. Combine the two subtables into a new table and run the logistic regression (with formula HA ~ exposure + additional predictors, if any) using the data of this new table.
5. Save resulting model coefficients and variance-covariance matrix.
6. Start over at Step 3 and repeat the procedure for a certain number of iterations, e.g., 500.
7. After a sufficiently large number of iterations of the above steps, the average exposure-response relationship for a cutoff of 72.73% can be simply obtained from the means of the 500 resulting model coefficient sets; in addition, confidence intervals can be calculated from the saved variance-covariance matrices.

There are several slightly different computational approaches to implement the above procedure. As a practical guide, in the Supplementary Materials, readers can find a generic script in the statistical programming language R (plus a sample data file) that calculates the simulated coefficients for a crude model and plots the corresponding exposure-response curve with confidence intervals. To account for correct confidence intervals around the simulated curve, the script considers the within-variance (variance within each calculated model), as well as the between-variance (variance of the coefficients between the 500 sampled models).

### 3.3. Which Value for F_above_ Is the ‘True’ One?

As described in the previous section, F_above_ is the crucial parameter to mimic the exposure-response relationship given a desired cutoff point as accurate as possible. Assuming an expectation-free, i.e. uniform distribution of responses in the “very” category to fall at any value between the lower and upper bound of the continuum covered by the “very” category, F_above_ takes the value 0.36 for a cutoff point at 72.73% (cf. Table 3). As shown above, this value is challenged by some (yet unsystematic) empirical findings and seems, on average, to be rather in the region of 0.5, at least in the surveys included in the present exercise (cf. Figure 4).

**Figure 5:**
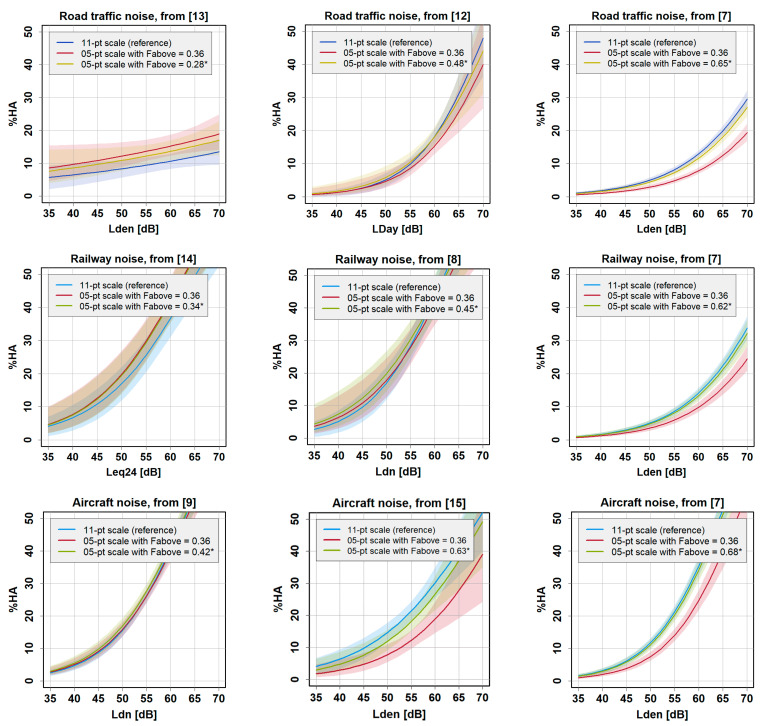
Exposure–response curves from different noise annoyance surveys showing %HA, as derived from the 11-point scale with cutoff point at 72.73% (“reference”), as well as based on the simulation with responses from the 5-point scale (with 95% confidence intervals as shaded areas). Two simulated curves are shown, one for F_above_ = 0.36 and one for the empirically derived F_above_ value of the respective study, marked with an asterisk (*). Curves are based on a simple unadjusted (crude) model. Data sources are from the following studies: [7,8,9,12,13,14,15].

For the 72.73% cutoff point, Figure 5 illustrates the difference between the exposure–HA curve for the expectation-free assumption of F_above_ (0.34) and the empirically derived value for F_above_ in each study whose distribution of values on the 11-point scale for the “very” annoyed is known. To draw the curves and confidence intervals, we employed the random sampling approach described above. Analyses were performed with R version 3.5.1.

Figure 5 shows that, expectedly, the empirically derived value assumed for F_above_ (light green curve) brings the simulated curves in almost all cases closer to the “reference” curve (blue curve) than does the value of 0.36 (red curve). This observation would, of course, challenge a recommendation to generally adopt 0.36. However, from Figure 5, we also learn that the empirical value of F_above_ can be smaller than 0.36. This makes it difficult to recommend using a particular value. Some considerations regarding that problem are discussed further below.

Researchers who want to use the R script (provided in the Supplementary Materials) with the expectation-free value of F_above_ for other conversions than the example discussed here should refer to Table 3.

## 4. Discussion

In this paper’s first part, we presented rules for converting annoyance response data that originate from different response scales to a unified scale from 0 to 100 points in order to be able to pool annoyance scores and compare resulting linear exposure–annoyance relationships. We could show that depending on the conversion rule applied, the resulting linear regression lines (annoyance score regressed on exposure) differ. For the conversion types “lower bound,” “midpoint,” and “upper bound,” as expected, the regression lines are simply shifted, while the “upscaling” conversion produces a steeper regression line in all three examples. We recommend using the upscale conversion for linear regression purposes due to its conservation of the slope that is observed with the original scale.

In the second part of this paper, we presented results of a simulation exercise for predicting the percentage of highly annoyed (%HA) based on response data on the 5-point scale; however, we used the non-native cutoff point of 72.73%, i.e., the value resulting from defining the three uppermost points on an 11-point numerical scale as indicating high annoyance (HA). We provided the necessary program code in the R language in the Supplementary Materials section for researchers to be able to reproduce and adapt the exercise if desired.

It turned out that the crucial parameter for the as-accurate-as-possible simulation of the HA exposure-response relationships for an arbitrary cutoff point (in the present example, 72.73%) is the fraction of respondents that are regarded as being above the cutoff point. In other words, for different assumptions of F_above_, different exposure-response relationships for %HA were obtained. While the expectation-free (“theoretical”) value of 0.36 assumes a uniform distribution of annoyance intensity within the “very” category, we could demonstrate that the empirically obtained values can deviate considerably from the theoretical expectation. In our sample of surveys, the empirical F_above_ values ranged from 0.28 to 0.68. It is thus generally not unproblematic to pool or compare the data from the two different scales. Our sample of surveys is probably too small and seems too heterogeneous to recommend a particular value for F_above_; however, there are signs that the survey average of F_above_ is probably larger than 0.36. The potential reasons for this remain elusive and cannot be examined within the scope of this exercise. Of course, a potentially relevant factor for the variability of F_above_ could be the language in which the annoyance questions and scale point labels are posed. In order to be able to more generally recommend a value for F_above_, more surveys (that have used both the 5-point and 11-point scales) would be needed. However, such an undertaking would still be quite difficult due to the paucity of available data/surveys at hand.

The apparent heterogeneity of the empirically derived F_above_ values also puts the seminal meta-analysis of Miedema and Vos [6] in a different light because those two authors assumed, for reasons of simplicity, that the annoyance intensity is uniformly distributed between the lower and upper bounds of a discrete category. Should F_above_ be systematically larger than the value a uniform distribution would imply, would the EU curves [16], in fact, have underestimated %HA. However, this must remain speculative. Our preliminary recommendation is therefore, to adhere to an F_above_ value that requires the least theoretical or empirical assumptions, i.e., the one assuming a uniform distribution. Of course, we do not discourage researchers from adopting another (higher) value based on the insights provided in the exercise presented above.

A more fundamental question is whether the 11-point numerical scale and hence the arbitrary cutoff point of 72.73% is still reasonable and recommendable to assess HA. It is noteworthy that in [2], in which the use of the 5-point verbal and the 11-point numerical scales were introduced as a quasi-standard, the ICBEN did not recommend how to define HA on the 11-point scale but did so only for the 5-point scale. The authors explained that in the psychometric study underlying the development of the original recommendation, the word “very” was the closest among several candidates to the word “highly” and that it was thus recommended that “very” together with “extremely” be used to define “high” annoyance, which resulted in a cutoff point of 60%. This cutoff point also encompassed such words as “considerably” (62% of the scale length), “substantially” (64% of the scale length), and “importantly” (65% of the scale length), “all of which indicate that the recommended high annoyance division identifies levels of annoyance that are not regarded as being trivial or moderate” ([2], p. 664). So, due to the lack of a clear (empirical) basis for a division of the 11-point numerical scale into “not highly annoyed” and “highly annoyed,” no cutoff point was recommended for that scale. The main aspect here is, that taking the verbal judgments of survey respondents as serious, is clearly less arbitrary than the setting of a numerical cutoff point. Therefore, for future research, we propose to extend the classical ICBEN recommendations beyond the choice of scale to the utilization of an empirically more robust definition of HA, i.e., the 5-point verbal scale and its 60% cutoff point. Comparisons with older 11-point data are still possible because, luckily, the simulation procedure described above works both ways and also allows for simulating the 60% cutoff point with response data on the 11-point scale.

## 5. Conclusions

The considerations in this paper extend the recommendations of the recently updated ISO standard [3] and describe how to compare and combine annoyance responses from the 5-point verbal and 11-point numerical scales for pooled analyses. Furthermore, the paper explains how to computationally simulate an exposure-HA relationship for any arbitrary cutoff point, regardless of the type of scale on which annoyance responses were collected.

## Figures and Tables

**Table 1:** Number of times either the 5-point, 11-point, or both annoyance scales, as well as other scale types, were used in the surveys included in the meta-analysis by Guski et al. [1].

Noise Source	11-Point Only	5-Point Only	Both 11-Point and 5-Point	Other Scales	Sum
Road traffic	8	3	7	7 ^a^	25
Railway	1	4	4	1 ^b^	10
Aircraft	9	1	5		15
Wind turbines	0	0	1	1 ^c^	2
Combined sources	1	1	3		5
Totals	19	9	20	9	57

^a^ 11-point and 4-point (one study); 4-point with notice filter question (six studies); ^b^ 11-point and 4-point; ^c^ 4-point and notice filter question.

**Table 2:** Conversions of scale point values on 4-, 5-, 6-, 7- and 11-point scales to values on an absolute annoyance intensity scale ranging from 0 to 100, rounded to two decimals.

**4-Point Numerical Scale**											
Numeric value:	0		1			2				3	
Upscaled value:	0		33			67				100	
Lower bound:	0.00		25.00			50.00				75.00	
Midpoint:	12.50		37.50			62.50				87.50	
Upper bound:	25.00		50.00			75.00				100.00	
				
**5-Point Verbal Scale (ICBEN Scale)**											
Scale point label:	“Not at all”		“Slightly”			“Moderately”		“Very”		“Extremely”	
Numeric value:	0		1			2		3		4	
Upscaled value:	0		25			50		75		100	
Lower bound:	0		20			40		60		80	
Midpoint:	10		30			50		70		90	
Upper bound:	20		40			60		80		100	
				
**6-Point Numerical Scale**											
Numeric value:	0		1	2		3		4		5	
Upscaled value:	0		20	40		60		80		100	
Lower bound:	0.00		16.67	33.33		50.00		66.67		83.33	
Midpoint:	8.33		25.00	41.67		58.33		75.00		91.67	
Upper bound:	16.67		33.33	50.00		66.67		83.33		100.00	
				
**7-Point Numerical Scale**											
Numerical value:	0	1	2	3		4		5		6	
Upscaled value:	0	17	33	50		67		83		100	
Lower bound:	0.00	14.29	28.57	42.86		57.14		71.43		85.71	
Midpoint:	7.14	21.43	35.71	50.00		64.29		78.57		92.86	
Upper bound:	14.29	28.57	42.86	57.14		71.43		85.71		100.00	
				
**11-Point Numerical Scale (ICBEN Scale)**											
Scale point label:	“Not at all”										“Extr.”
Numeric value:	0	1	2	3	4	5	6	7	8	9	10
Upscaled value:	0	10	20	30	40	50	60	70	80	90	100
Lower bound:	0.00	9.09	18.18	27.27	36.36	45.45	54.55	63.64	72.73	81.82	90.91
Midpoint:	4.55	13.64	22.73	31.82	40.90	50.00	59.09	68.18	77.27	86.36	95.50
Upper bound:	9.09	18.18	27.27	36.36	45.45	54.55	63.64	72.73	81.82	90.91	100.00

**Table 3:** F_below_ and F_above_ for different scales and cutoff points for a uniform and expectation-free distribution of the annoyance score (annoyance intensity) value within the respective category.

Scale	Desired Cutoff Point	Cutoff Point is in Category	F_below_	F_above_
5-point	60%	“very”/3	0.00	1.00
5-point	72%	“very”/3	0.60	0.40
5-point	72.73%	“very”/3	0.64	0.36
11-point	60%	“6”/6	0.60	0.40
11-point	72%	“7”/7	0.92	0.08
11-point	72.73%	“8”/8	0.00	1.00

## Data Availability

One part of the data that support the findings of this study are available from the corresponding author, upon reasonable request. Another part is available upon request from the Socio-Acoustic Survey Data Archive (SASDA) at I-INCE Japan. Further data illustrating the statistical exercise in this article can be found in the Supplementary Materials (File S1).

## Supplementary Materials

The following are available online at https://www.mdpi.com/article/10.3390/ijerph18147339/s1: File S1 includes a sample data file and a script in the R language demonstrating how to calculate and plot an exposure-response curve based on annoyance responses on a 5-point scale for a simulated cutoff point of 72.73%.
